# Supplementary material for: Classification of melanonychia, Beau’s lines, and nail clubbing based on nail images and transfer learning techniques
Source: PeerJ Comput Sci. 2023 Aug 24;9:e1533. doi: 10.7717/peerj-cs.1533 (PMC10495933; doi:10.7717/peerj-cs.1533)
Supplement: Supplemental Information 1 [file peerj-cs-09-1533-s001.zip › Codes and Console Outputs Supplementary Files/Code and Console Outputs for VGG16/ipythonVGG16Console output.html]

 

Epoch 15/30

3/3 [==============================] - 0s 44ms/step - loss: 0.2057 - accuracy: 0.9583 - val\_loss: 0.3007 - val\_accuracy: 0.8684

Epoch 16/30

3/3 [==============================] - 0s 42ms/step - loss: 0.1864 - accuracy: 0.9673 - val\_loss: 0.2906 - val\_accuracy: 0.8947

Epoch 17/30

3/3 [==============================] - 0s 42ms/step - loss: 0.1736 - accuracy: 0.9732 - val\_loss: 0.2791 - val\_accuracy: 0.8947

Epoch 18/30

3/3 [==============================] - 0s 42ms/step - loss: 0.1580 - accuracy: 0.9762 - val\_loss: 0.2704 - val\_accuracy: 0.8947

Epoch 19/30

3/3 [==============================] - 0s 42ms/step - loss: 0.1497 - accuracy: 0.9792 - val\_loss: 0.2532 - val\_accuracy: 0.8947

Epoch 20/30

3/3 [==============================] - 0s 42ms/step - loss: 0.1380 - accuracy: 0.9911 - val\_loss: 0.2442 - val\_accuracy: 0.9211

Epoch 21/30

3/3 [==============================] - 0s 41ms/step - loss: 0.1281 - accuracy: 0.9970 - val\_loss: 0.2330 - val\_accuracy: 0.8947

Epoch 22/30

3/3 [==============================] - 0s 42ms/step - loss: 0.1180 - accuracy: 0.9851 - val\_loss: 0.2257 - val\_accuracy: 0.8947

Epoch 23/30

3/3 [==============================] - 0s 42ms/step - loss: 0.1099 - accuracy: 0.9881 - val\_loss: 0.2167 - val\_accuracy: 0.9211

Epoch 24/30

3/3 [==============================] - 0s 26ms/step - loss: 0.1037 - accuracy: 1.0000 - val\_loss: 0.2120 - val\_accuracy: 0.9211

Epoch 25/30

3/3 [==============================] - 0s 29ms/step - loss: 0.0958 - accuracy: 1.0000 - val\_loss: 0.2099 - val\_accuracy: 0.8947

Epoch 26/30

3/3 [==============================] - 0s 41ms/step - loss: 0.0911 - accuracy: 0.9881 - val\_loss: 0.2025 - val\_accuracy: 0.9211

Epoch 27/30

3/3 [==============================] - 0s 42ms/step - loss: 0.0840 - accuracy: 0.9970 - val\_loss: 0.1977 - val\_accuracy: 0.9211

Epoch 28/30

3/3 [==============================] - 0s 27ms/step - loss: 0.0803 - accuracy: 1.0000 - val\_loss: 0.1915 - val\_accuracy: 0.9211

Epoch 29/30

3/3 [==============================] - 0s 43ms/step - loss: 0.0750 - accuracy: 1.0000 - val\_loss: 0.1871 - val\_accuracy: 0.9211

Epoch 30/30

3/3 [==============================] - 0s 44ms/step - loss: 0.0709 - accuracy: 1.0000 - val\_loss: 0.1845 - val\_accuracy: 0.9211

Epoch 1/30

3/3 [==============================] - 2s 274ms/step - loss: 1.9800 - accuracy: 0.3482 - val\_loss: 1.2510 - val\_accuracy: 0.2105

Epoch 2/30

3/3 [==============================] - 0s 41ms/step - loss: 1.1762 - accuracy: 0.3899 - val\_loss: 1.1626 - val\_accuracy: 0.3158

Epoch 3/30

3/3 [==============================] - 0s 36ms/step - loss: 0.9561 - accuracy: 0.4196 - val\_loss: 0.7863 - val\_accuracy: 0.7368

Epoch 4/30

3/3 [==============================] - 0s 28ms/step - loss: 0.7485 - accuracy: 0.6339 - val\_loss: 0.6878 - val\_accuracy: 0.7105

Epoch 5/30

3/3 [==============================] - 0s 31ms/step - loss: 0.6726 - accuracy: 0.7589 - val\_loss: 0.6590 - val\_accuracy: 0.7632

Epoch 6/30

3/3 [==============================] - 0s 32ms/step - loss: 0.5680 - accuracy: 0.8274 - val\_loss: 0.6172 - val\_accuracy: 0.7895

Epoch 7/30

3/3 [==============================] - 0s 38ms/step - loss: 0.4834 - accuracy: 0.8631 - val\_loss: 0.6301 - val\_accuracy: 0.7632

Epoch 8/30

3/3 [==============================] - 0s 30ms/step - loss: 0.4629 - accuracy: 0.8631 - val\_loss: 0.5433 - val\_accuracy: 0.8158

Epoch 9/30

3/3 [==============================] - 0s 30ms/step - loss: 0.3912 - accuracy: 0.8899 - val\_loss: 0.4923 - val\_accuracy: 0.8421

Epoch 10/30

3/3 [==============================] - 0s 28ms/step - loss: 0.3641 - accuracy: 0.8810 - val\_loss: 0.4750 - val\_accuracy: 0.8158

Epoch 11/30

3/3 [==============================] - 0s 29ms/step - loss: 0.3293 - accuracy: 0.8899 - val\_loss: 0.4570 - val\_accuracy: 0.8684

Epoch 12/30

3/3 [==============================] - 0s 29ms/step - loss: 0.3007 - accuracy: 0.9315 - val\_loss: 0.4651 - val\_accuracy: 0.9211

Epoch 13/30

3/3 [==============================] - 0s 32ms/step - loss: 0.2832 - accuracy: 0.9405 - val\_loss: 0.4401 - val\_accuracy: 0.8947

Epoch 14/30

3/3 [==============================] - 0s 43ms/step - loss: 0.2579 - accuracy: 0.9405 - val\_loss: 0.4177 - val\_accuracy: 0.8421

Epoch 15/30

3/3 [==============================] - 0s 29ms/step - loss: 0.2448 - accuracy: 0.9286 - val\_loss: 0.3972 - val\_accuracy: 0.8421

Epoch 16/30

3/3 [==============================] - 0s 29ms/step - loss: 0.2254 - accuracy: 0.9494 - val\_loss: 0.3932 - val\_accuracy: 0.8947

Epoch 17/30

3/3 [==============================] - 0s 38ms/step - loss: 0.2095 - accuracy: 0.9583 - val\_loss: 0.3889 - val\_accuracy: 0.8947

Epoch 18/30

3/3 [==============================] - 0s 59ms/step - loss: 0.1980 - accuracy: 0.9643 - val\_loss: 0.3753 - val\_accuracy: 0.8947

Epoch 19/30

3/3 [==============================] - 0s 28ms/step - loss: 0.1835 - accuracy: 0.9673 - val\_loss: 0.3627 - val\_accuracy: 0.8947

Epoch 20/30

3/3 [==============================] - 0s 28ms/step - loss: 0.1762 - accuracy: 0.9643 - val\_loss: 0.3540 - val\_accuracy: 0.8684

Epoch 21/30

3/3 [==============================] - 0s 29ms/step - loss: 0.1657 - accuracy: 0.9702 - val\_loss: 0.3458 - val\_accuracy: 0.8947

Epoch 22/30

3/3 [==============================] - 0s 28ms/step - loss: 0.1569 - accuracy: 0.9821 - val\_loss: 0.3470 - val\_accuracy: 0.8947

Epoch 23/30

3/3 [==============================] - 0s 26ms/step - loss: 0.1482 - accuracy: 0.9851 - val\_loss: 0.3368 - val\_accuracy: 0.8947

Epoch 24/30

3/3 [==============================] - 0s 30ms/step - loss: 0.1396 - accuracy: 0.9851 - val\_loss: 0.3269 - val\_accuracy: 0.8684

Epoch 25/30

3/3 [==============================] - 0s 29ms/step - loss: 0.1346 - accuracy: 0.9732 - val\_loss: 0.3164 - val\_accuracy: 0.8684

Epoch 26/30

3/3 [==============================] - 0s 29ms/step - loss: 0.1260 - accuracy: 0.9851 - val\_loss: 0.3083 - val\_accuracy: 0.8947

Epoch 27/30

3/3 [==============================] - 0s 32ms/step - loss: 0.1199 - accuracy: 0.9851 - val\_loss: 0.3077 - val\_accuracy: 0.8947

Epoch 28/30

3/3 [==============================] - 0s 31ms/step - loss: 0.1164 - accuracy: 0.9851 - val\_loss: 0.3057 - val\_accuracy: 0.8947

Epoch 29/30

3/3 [==============================] - 0s 35ms/step - loss: 0.1092 - accuracy: 0.9881 - val\_loss: 0.2962 - val\_accuracy: 0.8947

Epoch 30/30

3/3 [==============================] - 0s 27ms/step - loss: 0.1044 - accuracy: 0.9911 - val\_loss: 0.2905 - val\_accuracy: 0.8947

Epoch 1/30

3/3 [==============================] - 1s 222ms/step - loss: 1.7308 - accuracy: 0.3274 - val\_loss: 1.0578 - val\_accuracy: 0.4211

Epoch 2/30

3/3 [==============================] - 0s 76ms/step - loss: 1.0313 - accuracy: 0.4643 - val\_loss: 1.1033 - val\_accuracy: 0.4211

Epoch 3/30

3/3 [==============================] - 0s 30ms/step - loss: 0.7169 - accuracy: 0.6518 - val\_loss: 0.6153 - val\_accuracy: 0.6842

Epoch 4/30

3/3 [==============================] - 0s 60ms/step - loss: 0.5627 - accuracy: 0.7351 - val\_loss: 0.6169 - val\_accuracy: 0.6842

Epoch 5/30

3/3 [==============================] - 0s 34ms/step - loss: 0.4661 - accuracy: 0.8452 - val\_loss: 0.5491 - val\_accuracy: 0.8421

Epoch 6/30

3/3 [==============================] - 0s 27ms/step - loss: 0.3930 - accuracy: 0.8750 - val\_loss: 0.4790 - val\_accuracy: 0.8421

Epoch 7/30

3/3 [==============================] - 0s 27ms/step - loss: 0.3315 - accuracy: 0.9048 - val\_loss: 0.4412 - val\_accuracy: 0.7632

Epoch 8/30

3/3 [==============================] - 0s 27ms/step - loss: 0.2847 - accuracy: 0.9345 - val\_loss: 0.4453 - val\_accuracy: 0.7632

Epoch 9/30

3/3 [==============================] - 0s 28ms/step - loss: 0.2405 - accuracy: 0.9315 - val\_loss: 0.3717 - val\_accuracy: 0.8421

Epoch 10/30

3/3 [==============================] - 0s 26ms/step - loss: 0.2182 - accuracy: 0.9405 - val\_loss: 0.3195 - val\_accuracy: 0.8684

Epoch 11/30

3/3 [==============================] - 0s 27ms/step - loss: 0.1897 - accuracy: 0.9435 - val\_loss: 0.2961 - val\_accuracy: 0.9211

Epoch 12/30

3/3 [==============================] - 0s 27ms/step - loss: 0.1640 - accuracy: 0.9554 - val\_loss: 0.2665 - val\_accuracy: 0.9211

Epoch 13/30

3/3 [==============================] - 0s 26ms/step - loss: 0.1455 - accuracy: 0.9702 - val\_loss: 0.2235 - val\_accuracy: 0.9211

Epoch 14/30

3/3 [==============================] - 0s 27ms/step - loss: 0.1279 - accuracy: 0.9762 - val\_loss: 0.2159 - val\_accuracy: 0.9211

Epoch 15/30

3/3 [==============================] - 0s 27ms/step - loss: 0.1178 - accuracy: 0.9762 - val\_loss: 0.2030 - val\_accuracy: 0.9474

Epoch 16/30

3/3 [==============================] - 0s 28ms/step - loss: 0.1065 - accuracy: 0.9762 - val\_loss: 0.2017 - val\_accuracy: 0.9474

Epoch 17/30

3/3 [==============================] - 0s 26ms/step - loss: 0.0987 - accuracy: 0.9792 - val\_loss: 0.1888 - val\_accuracy: 0.9474

Epoch 18/30

3/3 [==============================] - 0s 27ms/step - loss: 0.0889 - accuracy: 0.9851 - val\_loss: 0.1764 - val\_accuracy: 0.9474

Epoch 19/30

3/3 [==============================] - 0s 26ms/step - loss: 0.0827 - accuracy: 0.9881 - val\_loss: 0.1740 - val\_accuracy: 0.9474

Epoch 20/30

3/3 [==============================] - 0s 26ms/step - loss: 0.0761 - accuracy: 0.9881 - val\_loss: 0.1756 - val\_accuracy: 0.9474

Epoch 21/30

3/3 [==============================] - 0s 27ms/step - loss: 0.0704 - accuracy: 0.9911 - val\_loss: 0.1708 - val\_accuracy: 0.9474

Epoch 22/30

3/3 [==============================] - 0s 27ms/step - loss: 0.0672 - accuracy: 0.9940 - val\_loss: 0.1571 - val\_accuracy: 0.9474

Epoch 23/30

3/3 [==============================] - 0s 27ms/step - loss: 0.0617 - accuracy: 0.9940 - val\_loss: 0.1628 - val\_accuracy: 0.9474

Epoch 24/30

3/3 [==============================] - 0s 27ms/step - loss: 0.0583 - accuracy: 0.9940 - val\_loss: 0.1469 - val\_accuracy: 0.9474

Epoch 25/30

3/3 [==============================] - 0s 26ms/step - loss: 0.0535 - accuracy: 0.9940 - val\_loss: 0.1389 - val\_accuracy: 0.9474

Epoch 26/30

3/3 [==============================] - 0s 27ms/step - loss: 0.0510 - accuracy: 0.9970 - val\_loss: 0.1450 - val\_accuracy: 0.9474

Epoch 27/30

3/3 [==============================] - 0s 26ms/step - loss: 0.0472 - accuracy: 0.9970 - val\_loss: 0.1408 - val\_accuracy: 0.9474

Epoch 28/30

3/3 [==============================] - 0s 27ms/step - loss: 0.0444 - accuracy: 0.9970 - val\_loss: 0.1404 - val\_accuracy: 0.9474

Epoch 29/30

3/3 [==============================] - 0s 26ms/step - loss: 0.0418 - accuracy: 0.9970 - val\_loss: 0.1288 - val\_accuracy: 0.9474

Epoch 30/30

3/3 [==============================] - 0s 26ms/step - loss: 0.0396 - accuracy: 0.9970 - val\_loss: 0.1240 - val\_accuracy: 0.9737

Epoch 1/30

3/3 [==============================] - 1s 343ms/step - loss: 1.3094 - accuracy: 0.4077 - val\_loss: 1.3158 - val\_accuracy: 0.4474

Epoch 2/30

3/3 [==============================] - 0s 37ms/step - loss: 0.8910 - accuracy: 0.6220 - val\_loss: 0.8748 - val\_accuracy: 0.6316

Epoch 3/30

3/3 [==============================] - 0s 27ms/step - loss: 0.6466 - accuracy: 0.6637 - val\_loss: 0.6798 - val\_accuracy: 0.6842

Epoch 4/30

3/3 [==============================] - 0s 26ms/step - loss: 0.4273 - accuracy: 0.8690 - val\_loss: 0.6723 - val\_accuracy: 0.8158

Epoch 5/30

3/3 [==============================] - 0s 27ms/step - loss: 0.3843 - accuracy: 0.8423 - val\_loss: 0.7131 - val\_accuracy: 0.8158

Epoch 6/30

3/3 [==============================] - 0s 28ms/step - loss: 0.2749 - accuracy: 0.9196 - val\_loss: 0.7102 - val\_accuracy: 0.7895

Epoch 7/30

3/3 [==============================] - 0s 26ms/step - loss: 0.2448 - accuracy: 0.9137 - val\_loss: 0.6941 - val\_accuracy: 0.7632

Epoch 8/30

3/3 [==============================] - 0s 27ms/step - loss: 0.2014 - accuracy: 0.9345 - val\_loss: 0.6883 - val\_accuracy: 0.8158

Epoch 9/30

3/3 [==============================] - 0s 28ms/step - loss: 0.1611 - accuracy: 0.9613 - val\_loss: 0.7310 - val\_accuracy: 0.8158

Epoch 10/30

3/3 [==============================] - 0s 26ms/step - loss: 0.1409 - accuracy: 0.9643 - val\_loss: 0.7072 - val\_accuracy: 0.8158

Epoch 11/30

3/3 [==============================] - 0s 26ms/step - loss: 0.1135 - accuracy: 0.9792 - val\_loss: 0.6934 - val\_accuracy: 0.8158

Epoch 12/30

3/3 [==============================] - 0s 27ms/step - loss: 0.1040 - accuracy: 0.9762 - val\_loss: 0.6982 - val\_accuracy: 0.8158

Epoch 13/30

3/3 [==============================] - 0s 26ms/step - loss: 0.0867 - accuracy: 0.9851 - val\_loss: 0.7118 - val\_accuracy: 0.8158

Epoch 14/30

3/3 [==============================] - 0s 26ms/step - loss: 0.0775 - accuracy: 0.9911 - val\_loss: 0.7059 - val\_accuracy: 0.8421

Epoch 15/30

3/3 [==============================] - 0s 26ms/step - loss: 0.0720 - accuracy: 0.9911 - val\_loss: 0.6853 - val\_accuracy: 0.8421

Epoch 16/30

3/3 [==============================] - 0s 26ms/step - loss: 0.0644 - accuracy: 0.9911 - val\_loss: 0.6852 - val\_accuracy: 0.8158

Epoch 17/30

3/3 [==============================] - 0s 26ms/step - loss: 0.0588 - accuracy: 0.9940 - val\_loss: 0.6805 - val\_accuracy: 0.8158

Epoch 18/30

3/3 [==============================] - 0s 26ms/step - loss: 0.0547 - accuracy: 0.9940 - val\_loss: 0.6711 - val\_accuracy: 0.8421

Epoch 19/30

3/3 [==============================] - 0s 26ms/step - loss: 0.0498 - accuracy: 0.9970 - val\_loss: 0.6944 - val\_accuracy: 0.8421

Epoch 20/30

3/3 [==============================] - 0s 26ms/step - loss: 0.0465 - accuracy: 0.9940 - val\_loss: 0.7124 - val\_accuracy: 0.8421

Epoch 21/30

3/3 [==============================] - 0s 27ms/step - loss: 0.0433 - accuracy: 0.9940 - val\_loss: 0.6909 - val\_accuracy: 0.8421

Epoch 22/30

3/3 [==============================] - 0s 26ms/step - loss: 0.0407 - accuracy: 0.9970 - val\_loss: 0.6782 - val\_accuracy: 0.8421

Epoch 23/30

3/3 [==============================] - 0s 26ms/step - loss: 0.0379 - accuracy: 0.9970 - val\_loss: 0.6996 - val\_accuracy: 0.8421

Epoch 24/30

3/3 [==============================] - 0s 26ms/step - loss: 0.0358 - accuracy: 0.9970 - val\_loss: 0.7037 - val\_accuracy: 0.8421

Epoch 25/30

3/3 [==============================] - 0s 26ms/step - loss: 0.0334 - accuracy: 0.9970 - val\_loss: 0.7057 - val\_accuracy: 0.8421

Epoch 26/30

3/3 [==============================] - 0s 30ms/step - loss: 0.0317 - accuracy: 0.9970 - val\_loss: 0.7035 - val\_accuracy: 0.8421

Epoch 27/30

3/3 [==============================] - 0s 26ms/step - loss: 0.0300 - accuracy: 0.9970 - val\_loss: 0.7010 - val\_accuracy: 0.8421

Epoch 28/30

3/3 [==============================] - 0s 25ms/step - loss: 0.0284 - accuracy: 0.9970 - val\_loss: 0.6989 - val\_accuracy: 0.8421

Epoch 29/30

3/3 [==============================] - 0s 25ms/step - loss: 0.0269 - accuracy: 0.9970 - val\_loss: 0.6968 - val\_accuracy: 0.8421

Epoch 30/30

3/3 [==============================] - 0s 26ms/step - loss: 0.0258 - accuracy: 0.9970 - val\_loss: 0.6969 - val\_accuracy: 0.8421

Epoch 1/30

3/3 [==============================] - 1s 211ms/step - loss: 1.5234 - accuracy: 0.4256 - val\_loss: 1.3380 - val\_accuracy: 0.6053

Epoch 2/30

3/3 [==============================] - 0s 36ms/step - loss: 0.8623 - accuracy: 0.6131 - val\_loss: 0.9126 - val\_accuracy: 0.4737

Epoch 3/30

3/3 [==============================] - 0s 26ms/step - loss: 0.6783 - accuracy: 0.6964 - val\_loss: 0.6167 - val\_accuracy: 0.7632

Epoch 4/30

3/3 [==============================] - 0s 27ms/step - loss: 0.4513 - accuracy: 0.8363 - val\_loss: 0.6765 - val\_accuracy: 0.6316

Epoch 5/30

3/3 [==============================] - 0s 27ms/step - loss: 0.3710 - accuracy: 0.8601 - val\_loss: 0.4286 - val\_accuracy: 0.8947

Epoch 6/30

3/3 [==============================] - 0s 26ms/step - loss: 0.2918 - accuracy: 0.9018 - val\_loss: 0.3832 - val\_accuracy: 0.8158

Epoch 7/30

3/3 [==============================] - 0s 27ms/step - loss: 0.2549 - accuracy: 0.9137 - val\_loss: 0.3504 - val\_accuracy: 0.9474

Epoch 8/30

3/3 [==============================] - 0s 26ms/step - loss: 0.2026 - accuracy: 0.9524 - val\_loss: 0.3289 - val\_accuracy: 0.8947

Epoch 9/30

3/3 [==============================] - 0s 27ms/step - loss: 0.1785 - accuracy: 0.9464 - val\_loss: 0.2891 - val\_accuracy: 0.9474

Epoch 10/30

3/3 [==============================] - 0s 26ms/step - loss: 0.1506 - accuracy: 0.9732 - val\_loss: 0.2466 - val\_accuracy: 0.9474

Epoch 11/30

3/3 [==============================] - 0s 27ms/step - loss: 0.1347 - accuracy: 0.9702 - val\_loss: 0.2227 - val\_accuracy: 0.9474

Epoch 12/30

3/3 [==============================] - 0s 27ms/step - loss: 0.1169 - accuracy: 0.9792 - val\_loss: 0.2182 - val\_accuracy: 0.9474

Epoch 13/30

3/3 [==============================] - 0s 26ms/step - loss: 0.1056 - accuracy: 0.9881 - val\_loss: 0.2295 - val\_accuracy: 0.9474

Epoch 14/30

3/3 [==============================] - 0s 27ms/step - loss: 0.0964 - accuracy: 0.9881 - val\_loss: 0.2106 - val\_accuracy: 0.9474

Epoch 15/30

3/3 [==============================] - 0s 27ms/step - loss: 0.0847 - accuracy: 0.9851 - val\_loss: 0.1919 - val\_accuracy: 0.9474

Epoch 16/30

3/3 [==============================] - 0s 26ms/step - loss: 0.0781 - accuracy: 0.9911 - val\_loss: 0.1846 - val\_accuracy: 0.9474

Epoch 17/30

3/3 [==============================] - 0s 26ms/step - loss: 0.0708 - accuracy: 0.9940 - val\_loss: 0.1830 - val\_accuracy: 0.9474

Epoch 18/30

3/3 [==============================] - 0s 26ms/step - loss: 0.0653 - accuracy: 0.9970 - val\_loss: 0.1928 - val\_accuracy: 0.9474

Epoch 19/30

3/3 [==============================] - 0s 27ms/step - loss: 0.0595 - accuracy: 1.0000 - val\_loss: 0.1861 - val\_accuracy: 0.9474

Epoch 20/30

3/3 [==============================] - 0s 27ms/step - loss: 0.0537 - accuracy: 1.0000 - val\_loss: 0.1745 - val\_accuracy: 0.9474

Epoch 21/30

3/3 [==============================] - 0s 28ms/step - loss: 0.0497 - accuracy: 1.0000 - val\_loss: 0.1695 - val\_accuracy: 0.9474

Epoch 22/30

3/3 [==============================] - 0s 28ms/step - loss: 0.0458 - accuracy: 1.0000 - val\_loss: 0.1744 - val\_accuracy: 0.9474

Epoch 23/30

3/3 [==============================] - 0s 28ms/step - loss: 0.0428 - accuracy: 1.0000 - val\_loss: 0.1747 - val\_accuracy: 0.9474

Epoch 24/30

3/3 [==============================] - 0s 27ms/step - loss: 0.0398 - accuracy: 1.0000 - val\_loss: 0.1710 - val\_accuracy: 0.9474

Epoch 25/30

3/3 [==============================] - 0s 27ms/step - loss: 0.0370 - accuracy: 1.0000 - val\_loss: 0.1729 - val\_accuracy: 0.9474

Epoch 26/30

3/3 [==============================] - 0s 27ms/step - loss: 0.0344 - accuracy: 1.0000 - val\_loss: 0.1740 - val\_accuracy: 0.9474

Epoch 27/30

3/3 [==============================] - 0s 27ms/step - loss: 0.0328 - accuracy: 1.0000 - val\_loss: 0.1675 - val\_accuracy: 0.9474

Epoch 28/30

3/3 [==============================] - 0s 25ms/step - loss: 0.0309 - accuracy: 1.0000 - val\_loss: 0.1550 - val\_accuracy: 0.9474

Epoch 29/30

3/3 [==============================] - 0s 26ms/step - loss: 0.0292 - accuracy: 1.0000 - val\_loss: 0.1592 - val\_accuracy: 0.9474

Epoch 30/30

3/3 [==============================] - 0s 26ms/step - loss: 0.0273 - accuracy: 1.0000 - val\_loss: 0.1734 - val\_accuracy: 0.9474

Epoch 1/30

3/3 [==============================] - 1s 253ms/step - loss: 1.2228 - accuracy: 0.3958 - val\_loss: 1.0564 - val\_accuracy: 0.3947

Epoch 2/30

3/3 [==============================] - 0s 47ms/step - loss: 0.8797 - accuracy: 0.5476 - val\_loss: 0.6931 - val\_accuracy: 0.7368

Epoch 3/30

3/3 [==============================] - 0s 25ms/step - loss: 0.6621 - accuracy: 0.6964 - val\_loss: 0.6904 - val\_accuracy: 0.6842

Epoch 4/30

3/3 [==============================] - 0s 25ms/step - loss: 0.5272 - accuracy: 0.7738 - val\_loss: 0.6062 - val\_accuracy: 0.6316

Epoch 5/30

3/3 [==============================] - 0s 25ms/step - loss: 0.4420 - accuracy: 0.8512 - val\_loss: 0.5927 - val\_accuracy: 0.6579

Epoch 6/30

3/3 [==============================] - 0s 41ms/step - loss: 0.3687 - accuracy: 0.8839 - val\_loss: 0.4222 - val\_accuracy: 0.8158

Epoch 7/30

3/3 [==============================] - 0s 41ms/step - loss: 0.3011 - accuracy: 0.8929 - val\_loss: 0.3889 - val\_accuracy: 0.9211

Epoch 8/30

3/3 [==============================] - 0s 41ms/step - loss: 0.2562 - accuracy: 0.9315 - val\_loss: 0.3334 - val\_accuracy: 0.8421

Epoch 9/30

3/3 [==============================] - 0s 41ms/step - loss: 0.2088 - accuracy: 0.9226 - val\_loss: 0.3025 - val\_accuracy: 0.8421

Epoch 10/30

3/3 [==============================] - 0s 41ms/step - loss: 0.1909 - accuracy: 0.9345 - val\_loss: 0.2600 - val\_accuracy: 0.9474

Epoch 11/30

3/3 [==============================] - 0s 26ms/step - loss: 0.1534 - accuracy: 0.9821 - val\_loss: 0.2549 - val\_accuracy: 0.9474

Epoch 12/30

3/3 [==============================] - 0s 28ms/step - loss: 0.1407 - accuracy: 0.9851 - val\_loss: 0.2164 - val\_accuracy: 0.9737

Epoch 13/30

3/3 [==============================] - 0s 42ms/step - loss: 0.1207 - accuracy: 0.9970 - val\_loss: 0.2023 - val\_accuracy: 0.9737

Epoch 14/30

3/3 [==============================] - 0s 41ms/step - loss: 0.1093 - accuracy: 0.9970 - val\_loss: 0.1882 - val\_accuracy: 0.9737

Epoch 15/30

3/3 [==============================] - 0s 42ms/step - loss: 0.0966 - accuracy: 0.9970 - val\_loss: 0.1801 - val\_accuracy: 0.9737

Epoch 16/30

3/3 [==============================] - 0s 39ms/step - loss: 0.0869 - accuracy: 1.0000 - val\_loss: 0.1778 - val\_accuracy: 0.9737

Epoch 17/30

3/3 [==============================] - 0s 41ms/step - loss: 0.0780 - accuracy: 0.9970 - val\_loss: 0.1624 - val\_accuracy: 0.9737

Epoch 18/30

3/3 [==============================] - 0s 42ms/step - loss: 0.0713 - accuracy: 0.9970 - val\_loss: 0.1578 - val\_accuracy: 0.9737

Epoch 19/30

3/3 [==============================] - 0s 41ms/step - loss: 0.0676 - accuracy: 0.9940 - val\_loss: 0.1546 - val\_accuracy: 0.9737

Epoch 20/30

3/3 [==============================] - 0s 41ms/step - loss: 0.0602 - accuracy: 1.0000 - val\_loss: 0.1621 - val\_accuracy: 0.9474

Epoch 21/30

3/3 [==============================] - 0s 41ms/step - loss: 0.0556 - accuracy: 1.0000 - val\_loss: 0.1493 - val\_accuracy: 0.9737

Epoch 22/30

3/3 [==============================] - 0s 41ms/step - loss: 0.0505 - accuracy: 1.0000 - val\_loss: 0.1423 - val\_accuracy: 0.9737

Epoch 23/30

3/3 [==============================] - 0s 41ms/step - loss: 0.0477 - accuracy: 0.9970 - val\_loss: 0.1434 - val\_accuracy: 0.9737

Epoch 24/30

3/3 [==============================] - 0s 40ms/step - loss: 0.0438 - accuracy: 1.0000 - val\_loss: 0.1465 - val\_accuracy: 0.9474

Epoch 25/30

3/3 [==============================] - 0s 44ms/step - loss: 0.0413 - accuracy: 1.0000 - val\_loss: 0.1460 - val\_accuracy: 0.9474

Epoch 26/30

3/3 [==============================] - 0s 44ms/step - loss: 0.0388 - accuracy: 1.0000 - val\_loss: 0.1368 - val\_accuracy: 0.9737

Epoch 27/30

3/3 [==============================] - 0s 51ms/step - loss: 0.0365 - accuracy: 1.0000 - val\_loss: 0.1354 - val\_accuracy: 0.9737

Epoch 28/30

3/3 [==============================] - 0s 42ms/step - loss: 0.0345 - accuracy: 1.0000 - val\_loss: 0.1417 - val\_accuracy: 0.9474

Epoch 29/30

3/3 [==============================] - 0s 42ms/step - loss: 0.0323 - accuracy: 1.0000 - val\_loss: 0.1375 - val\_accuracy: 0.9474

Epoch 30/30

3/3 [==============================] - 0s 57ms/step - loss: 0.0303 - accuracy: 1.0000 - val\_loss: 0.1346 - val\_accuracy: 0.9474

Epoch 1/30

3/3 [==============================] - 1s 155ms/step - loss: 1.2540 - accuracy: 0.4613 - val\_loss: 0.9072 - val\_accuracy: 0.4474

Epoch 2/30

3/3 [==============================] - 0s 26ms/step - loss: 0.7414 - accuracy: 0.6250 - val\_loss: 0.5673 - val\_accuracy: 0.6842

Epoch 3/30

3/3 [==============================] - 0s 27ms/step - loss: 0.5263 - accuracy: 0.7857 - val\_loss: 0.3976 - val\_accuracy: 0.8421

Epoch 4/30

3/3 [==============================] - 0s 27ms/step - loss: 0.4222 - accuracy: 0.8393 - val\_loss: 0.3934 - val\_accuracy: 0.8421

Epoch 5/30

3/3 [==============================] - 0s 27ms/step - loss: 0.2961 - accuracy: 0.8839 - val\_loss: 0.2687 - val\_accuracy: 0.9474

Epoch 6/30

3/3 [==============================] - 0s 26ms/step - loss: 0.2391 - accuracy: 0.9286 - val\_loss: 0.2848 - val\_accuracy: 0.8947

Epoch 7/30

3/3 [==============================] - 0s 26ms/step - loss: 0.1884 - accuracy: 0.9524 - val\_loss: 0.3343 - val\_accuracy: 0.8947

Epoch 8/30

3/3 [==============================] - 0s 25ms/step - loss: 0.1596 - accuracy: 0.9643 - val\_loss: 0.2496 - val\_accuracy: 0.9211

Epoch 9/30

3/3 [==============================] - 0s 26ms/step - loss: 0.1293 - accuracy: 0.9762 - val\_loss: 0.2483 - val\_accuracy: 0.9474

Epoch 10/30

3/3 [==============================] - 0s 27ms/step - loss: 0.1148 - accuracy: 0.9881 - val\_loss: 0.2516 - val\_accuracy: 0.8947

Epoch 11/30

3/3 [==============================] - 0s 24ms/step - loss: 0.0990 - accuracy: 0.9851 - val\_loss: 0.2056 - val\_accuracy: 0.9211

Epoch 12/30

3/3 [==============================] - 0s 26ms/step - loss: 0.0845 - accuracy: 0.9940 - val\_loss: 0.2131 - val\_accuracy: 0.9211

Epoch 13/30

3/3 [==============================] - 0s 27ms/step - loss: 0.0765 - accuracy: 0.9911 - val\_loss: 0.1961 - val\_accuracy: 0.9211

Epoch 14/30

3/3 [==============================] - 0s 26ms/step - loss: 0.0681 - accuracy: 0.9940 - val\_loss: 0.1645 - val\_accuracy: 0.9211

Epoch 15/30

3/3 [==============================] - 0s 63ms/step - loss: 0.0621 - accuracy: 0.9940 - val\_loss: 0.1658 - val\_accuracy: 0.9211

Epoch 16/30

3/3 [==============================] - 0s 26ms/step - loss: 0.0543 - accuracy: 0.9940 - val\_loss: 0.1643 - val\_accuracy: 0.9474

Epoch 17/30

3/3 [==============================] - 0s 25ms/step - loss: 0.0470 - accuracy: 0.9940 - val\_loss: 0.1357 - val\_accuracy: 0.9474

Epoch 18/30

3/3 [==============================] - 0s 26ms/step - loss: 0.0439 - accuracy: 0.9970 - val\_loss: 0.1349 - val\_accuracy: 0.9474

Epoch 19/30

3/3 [==============================] - 0s 28ms/step - loss: 0.0386 - accuracy: 0.9970 - val\_loss: 0.1439 - val\_accuracy: 0.9474

Epoch 20/30

3/3 [==============================] - 0s 27ms/step - loss: 0.0370 - accuracy: 0.9970 - val\_loss: 0.1362 - val\_accuracy: 0.9474

Epoch 21/30

3/3 [==============================] - 0s 26ms/step - loss: 0.0333 - accuracy: 0.9970 - val\_loss: 0.1194 - val\_accuracy: 0.9474

Epoch 22/30

3/3 [==============================] - 0s 27ms/step - loss: 0.0319 - accuracy: 1.0000 - val\_loss: 0.1213 - val\_accuracy: 0.9474

Epoch 23/30

3/3 [==============================] - 0s 26ms/step - loss: 0.0291 - accuracy: 1.0000 - val\_loss: 0.1275 - val\_accuracy: 0.9474

Epoch 24/30

3/3 [==============================] - 0s 25ms/step - loss: 0.0271 - accuracy: 1.0000 - val\_loss: 0.1235 - val\_accuracy: 0.9474

Epoch 25/30

3/3 [==============================] - 0s 25ms/step - loss: 0.0247 - accuracy: 1.0000 - val\_loss: 0.1178 - val\_accuracy: 0.9474

Epoch 26/30

3/3 [==============================] - 0s 25ms/step - loss: 0.0238 - accuracy: 1.0000 - val\_loss: 0.1153 - val\_accuracy: 0.9474

Epoch 27/30

3/3 [==============================] - 0s 25ms/step - loss: 0.0220 - accuracy: 1.0000 - val\_loss: 0.1174 - val\_accuracy: 0.9474

Epoch 28/30

3/3 [==============================] - 0s 25ms/step - loss: 0.0209 - accuracy: 1.0000 - val\_loss: 0.1171 - val\_accuracy: 0.9474

Epoch 29/30

3/3 [==============================] - 0s 24ms/step - loss: 0.0194 - accuracy: 1.0000 - val\_loss: 0.1200 - val\_accuracy: 0.9474

Epoch 30/30

3/3 [==============================] - 0s 25ms/step - loss: 0.0187 - accuracy: 1.0000 - val\_loss: 0.1232 - val\_accuracy: 0.9474

Accuracy : 0.9450980392156862

15/15 [==============================] - 25s 2s/step

8/8 [==============================] - 15s 2s/step

vgg16

You must install pydot (`pip install pydot`) and install graphviz (see instructions at https://graphviz.gitlab.io/download/) for plot\_model/model\_to\_dot to work.

Model: "model\_5"

\_\_\_\_\_\_\_\_\_\_\_\_\_\_\_\_\_\_\_\_\_\_\_\_\_\_\_\_\_\_\_\_\_\_\_\_\_\_\_\_\_\_\_\_\_\_\_\_\_\_\_\_\_\_\_\_\_\_\_\_\_\_\_\_\_

Layer (type) Output Shape Param #

=================================================================

input\_12 (InputLayer) [(None, 9, 9, 512)] 0

  

block5\_conv1 (Conv2D) multiple 2359808

  

block5\_conv2 (Conv2D) multiple 2359808

  

block5\_conv3 (Conv2D) multiple 2359808

  

block5\_pool (MaxPooling2D) multiple 0

  

conv2d\_12 (Conv2D) (None, 2, 2, 64) 294976

  

max\_pooling2d\_12 (MaxPoolin (None, 1, 1, 64) 0

g2D)

  

flatten\_40 (Flatten) (None, 64) 0

  

dense\_80 (Dense) (None, 100) 6500

  

dense\_81 (Dense) (None, 3) 303

  

=================================================================

Total params: 7,381,203

Trainable params: 7,381,203

Non-trainable params: 0

\_\_\_\_\_\_\_\_\_\_\_\_\_\_\_\_\_\_\_\_\_\_\_\_\_\_\_\_\_\_\_\_\_\_\_\_\_\_\_\_\_\_\_\_\_\_\_\_\_\_\_\_\_\_\_\_\_\_\_\_\_\_\_\_\_

Epoch 1/30

3/3 [==============================] - 9s 3s/step - loss: 1.1849 - acc: 0.3957 - val\_loss: 1.0681 - val\_acc: 0.4149

Epoch 2/30

3/3 [==============================] - 6s 2s/step - loss: 1.1047 - acc: 0.4144 - val\_loss: 0.8643 - val\_acc: 0.6915

Epoch 3/30

3/3 [==============================] - 7s 3s/step - loss: 0.8595 - acc: 0.6390 - val\_loss: 0.8838 - val\_acc: 0.5532

Epoch 4/30

3/3 [==============================] - 7s 2s/step - loss: 1.1536 - acc: 0.4385 - val\_loss: 0.9598 - val\_acc: 0.4043

Epoch 5/30

3/3 [==============================] - 7s 2s/step - loss: 0.8603 - acc: 0.5561 - val\_loss: 0.6990 - val\_acc: 0.6915

Epoch 6/30

3/3 [==============================] - 7s 2s/step - loss: 0.7421 - acc: 0.6658 - val\_loss: 0.5285 - val\_acc: 0.7553

Epoch 7/30

3/3 [==============================] - 6s 2s/step - loss: 0.6179 - acc: 0.7326 - val\_loss: 0.4512 - val\_acc: 0.8617

Epoch 8/30

3/3 [==============================] - 7s 2s/step - loss: 0.4575 - acc: 0.8235 - val\_loss: 0.4080 - val\_acc: 0.8511

Epoch 9/30

3/3 [==============================] - 7s 2s/step - loss: 0.3583 - acc: 0.8770 - val\_loss: 0.4154 - val\_acc: 0.8511

Epoch 10/30

3/3 [==============================] - 7s 3s/step - loss: 0.3146 - acc: 0.8717 - val\_loss: 0.3677 - val\_acc: 0.8404

Epoch 11/30

3/3 [==============================] - 7s 2s/step - loss: 0.2115 - acc: 0.9332 - val\_loss: 0.3181 - val\_acc: 0.8830

Epoch 12/30

3/3 [==============================] - 7s 2s/step - loss: 0.1919 - acc: 0.9278 - val\_loss: 0.3649 - val\_acc: 0.8936

Epoch 13/30

3/3 [==============================] - 7s 2s/step - loss: 0.1334 - acc: 0.9599 - val\_loss: 0.3361 - val\_acc: 0.8723

Epoch 14/30

3/3 [==============================] - 7s 2s/step - loss: 0.1050 - acc: 0.9599 - val\_loss: 0.3834 - val\_acc: 0.8723

Epoch 15/30

3/3 [==============================] - 7s 2s/step - loss: 0.0754 - acc: 0.9786 - val\_loss: 0.2983 - val\_acc: 0.9255

Epoch 16/30

3/3 [==============================] - 7s 2s/step - loss: 0.0525 - acc: 0.9840 - val\_loss: 0.3406 - val\_acc: 0.9149

Epoch 17/30

3/3 [==============================] - 7s 2s/step - loss: 0.0356 - acc: 0.9920 - val\_loss: 0.3291 - val\_acc: 0.9149

Epoch 18/30

3/3 [==============================] - 7s 2s/step - loss: 0.0152 - acc: 0.9920 - val\_loss: 0.3590 - val\_acc: 0.9362

Epoch 19/30

3/3 [==============================] - 7s 2s/step - loss: 0.0098 - acc: 0.9973 - val\_loss: 0.5911 - val\_acc: 0.9149

Epoch 20/30

3/3 [==============================] - 7s 2s/step - loss: 0.0051 - acc: 0.9973 - val\_loss: 0.5692 - val\_acc: 0.9362

Epoch 21/30

3/3 [==============================] - 7s 2s/step - loss: 0.0045 - acc: 0.9973 - val\_loss: 0.5989 - val\_acc: 0.9255

Epoch 22/30

3/3 [==============================] - 7s 3s/step - loss: 0.0911 - acc: 0.9786 - val\_loss: 0.6319 - val\_acc: 0.9255

Epoch 23/30

3/3 [==============================] - 7s 3s/step - loss: 0.2689 - acc: 0.9599 - val\_loss: 0.8716 - val\_acc: 0.8936

Epoch 24/30

3/3 [==============================] - 7s 3s/step - loss: 0.4149 - acc: 0.9091 - val\_loss: 0.6857 - val\_acc: 0.7872

Epoch 25/30

3/3 [==============================] - 7s 2s/step - loss: 0.4977 - acc: 0.8155 - val\_loss: 0.5013 - val\_acc: 0.8404

Epoch 26/30

3/3 [==============================] - 7s 2s/step - loss: 0.2752 - acc: 0.9278 - val\_loss: 0.6696 - val\_acc: 0.8511

Epoch 27/30

3/3 [==============================] - 7s 2s/step - loss: 0.1379 - acc: 0.9465 - val\_loss: 0.4081 - val\_acc: 0.8723

Epoch 28/30

3/3 [==============================] - 7s 2s/step - loss: 0.1074 - acc: 0.9545 - val\_loss: 0.5272 - val\_acc: 0.8511

Epoch 29/30

3/3 [==============================] - 7s 2s/step - loss: 0.0536 - acc: 0.9920 - val\_loss: 0.5893 - val\_acc: 0.8936

Epoch 30/30

3/3 [==============================] - 6s 2s/step - loss: 0.0276 - acc: 0.9920 - val\_loss: 0.5195 - val\_acc: 0.8936

new model

You must install pydot (`pip install pydot`) and install graphviz (see instructions at https://graphviz.gitlab.io/download/) for plot\_model/model\_to\_dot to work.

Fine Tuning Accuracy :

  

Accuracy : 0.9450980392156862

8/8 [==============================] - 2s 240ms/step - loss: 0.1099 - acc: 0.9804

\_\_\_\_\_\_\_\_\_\_\_\_\_\_\_\_\_\_\_\_\_\_\_\_\_\_\_\_\_\_\_\_\_\_

VGG16 Fine Tuning" :

\_\_\_\_\_\_\_\_\_\_\_\_\_\_\_\_\_\_\_\_\_\_\_\_\_\_\_\_\_\_\_\_\_\_\_\_\_

Model MAE MSE RMSE R2 Square Accuracy

0 VGG16 Fine Tuning 0.086275 0.14902 0.386031 0.773947 0.945098

In [8]:
