# Supplementary material for: Classification of melanonychia, Beau’s lines, and nail clubbing based on nail images and transfer learning techniques
Source: PeerJ Comput Sci. 2023 Aug 24;9:e1533. doi: 10.7717/peerj-cs.1533 (PMC10495933; doi:10.7717/peerj-cs.1533)
Supplement: Supplemental Information 1 [file peerj-cs-09-1533-s001.zip › Codes and Console Outputs Supplementary Files/Code and Console Outputs for VGG19/ipythonVGG19Console output.html]

 

Epoch 13/30

3/3 [==============================] - 0s 41ms/step - loss: 0.2838 - accuracy: 0.9375 - val\_loss: 0.4504 - val\_accuracy: 0.8684

Epoch 14/30

3/3 [==============================] - 0s 47ms/step - loss: 0.2646 - accuracy: 0.9435 - val\_loss: 0.4062 - val\_accuracy: 0.8684

Epoch 15/30

3/3 [==============================] - 0s 42ms/step - loss: 0.2397 - accuracy: 0.9554 - val\_loss: 0.3712 - val\_accuracy: 0.8684

Epoch 16/30

3/3 [==============================] - 0s 41ms/step - loss: 0.2273 - accuracy: 0.9673 - val\_loss: 0.3499 - val\_accuracy: 0.8947

Epoch 17/30

3/3 [==============================] - 0s 56ms/step - loss: 0.2126 - accuracy: 0.9643 - val\_loss: 0.3527 - val\_accuracy: 0.8947

Epoch 18/30

3/3 [==============================] - 0s 58ms/step - loss: 0.1996 - accuracy: 0.9702 - val\_loss: 0.3548 - val\_accuracy: 0.8947

Epoch 19/30

3/3 [==============================] - 0s 40ms/step - loss: 0.1895 - accuracy: 0.9673 - val\_loss: 0.3155 - val\_accuracy: 0.8947

Epoch 20/30

3/3 [==============================] - 0s 41ms/step - loss: 0.1792 - accuracy: 0.9732 - val\_loss: 0.2994 - val\_accuracy: 0.8947

Epoch 21/30

3/3 [==============================] - 0s 41ms/step - loss: 0.1695 - accuracy: 0.9702 - val\_loss: 0.3263 - val\_accuracy: 0.8947

Epoch 22/30

3/3 [==============================] - 0s 41ms/step - loss: 0.1621 - accuracy: 0.9732 - val\_loss: 0.3157 - val\_accuracy: 0.8947

Epoch 23/30

3/3 [==============================] - 0s 41ms/step - loss: 0.1517 - accuracy: 0.9792 - val\_loss: 0.2802 - val\_accuracy: 0.9211

Epoch 24/30

3/3 [==============================] - 0s 25ms/step - loss: 0.1458 - accuracy: 0.9821 - val\_loss: 0.3015 - val\_accuracy: 0.8947

Epoch 25/30

3/3 [==============================] - 0s 24ms/step - loss: 0.1380 - accuracy: 0.9851 - val\_loss: 0.3376 - val\_accuracy: 0.8947

Epoch 26/30

3/3 [==============================] - 0s 41ms/step - loss: 0.1324 - accuracy: 0.9851 - val\_loss: 0.2932 - val\_accuracy: 0.8947

Epoch 27/30

3/3 [==============================] - 0s 40ms/step - loss: 0.1268 - accuracy: 0.9881 - val\_loss: 0.2840 - val\_accuracy: 0.8947

Epoch 28/30

3/3 [==============================] - 0s 24ms/step - loss: 0.1183 - accuracy: 0.9881 - val\_loss: 0.3157 - val\_accuracy: 0.8947

Epoch 29/30

3/3 [==============================] - 0s 40ms/step - loss: 0.1148 - accuracy: 0.9881 - val\_loss: 0.3156 - val\_accuracy: 0.8947

Epoch 30/30

3/3 [==============================] - 0s 40ms/step - loss: 0.1087 - accuracy: 0.9881 - val\_loss: 0.2718 - val\_accuracy: 0.8947

Epoch 1/30

3/3 [==============================] - 1s 147ms/step - loss: 2.0909 - accuracy: 0.3690 - val\_loss: 1.4098 - val\_accuracy: 0.2368

Epoch 2/30

3/3 [==============================] - 0s 26ms/step - loss: 1.2120 - accuracy: 0.4077 - val\_loss: 0.9712 - val\_accuracy: 0.6842

Epoch 3/30

3/3 [==============================] - 0s 25ms/step - loss: 1.0946 - accuracy: 0.6042 - val\_loss: 0.7648 - val\_accuracy: 0.7105

Epoch 4/30

3/3 [==============================] - 0s 26ms/step - loss: 0.8062 - accuracy: 0.6220 - val\_loss: 0.6774 - val\_accuracy: 0.7368

Epoch 5/30

3/3 [==============================] - 0s 25ms/step - loss: 0.7051 - accuracy: 0.7202 - val\_loss: 0.7556 - val\_accuracy: 0.5789

Epoch 6/30

3/3 [==============================] - 0s 26ms/step - loss: 0.7014 - accuracy: 0.6280 - val\_loss: 0.6652 - val\_accuracy: 0.6842

Epoch 7/30

3/3 [==============================] - 0s 28ms/step - loss: 0.5984 - accuracy: 0.8571 - val\_loss: 0.5327 - val\_accuracy: 0.7368

Epoch 8/30

3/3 [==============================] - 0s 25ms/step - loss: 0.5591 - accuracy: 0.7411 - val\_loss: 0.5007 - val\_accuracy: 0.7632

Epoch 9/30

3/3 [==============================] - 0s 26ms/step - loss: 0.5240 - accuracy: 0.7470 - val\_loss: 0.5046 - val\_accuracy: 0.7368

Epoch 10/30

3/3 [==============================] - 0s 25ms/step - loss: 0.4786 - accuracy: 0.8393 - val\_loss: 0.4913 - val\_accuracy: 0.8421

Epoch 11/30

3/3 [==============================] - 0s 25ms/step - loss: 0.4440 - accuracy: 0.9077 - val\_loss: 0.4685 - val\_accuracy: 0.8421

Epoch 12/30

3/3 [==============================] - 0s 25ms/step - loss: 0.4176 - accuracy: 0.9048 - val\_loss: 0.4256 - val\_accuracy: 0.8947

Epoch 13/30

3/3 [==============================] - 0s 26ms/step - loss: 0.3858 - accuracy: 0.9226 - val\_loss: 0.4099 - val\_accuracy: 0.8158

Epoch 14/30

3/3 [==============================] - 0s 25ms/step - loss: 0.3661 - accuracy: 0.9256 - val\_loss: 0.3784 - val\_accuracy: 0.8421

Epoch 15/30

3/3 [==============================] - 0s 25ms/step - loss: 0.3398 - accuracy: 0.9435 - val\_loss: 0.3758 - val\_accuracy: 0.8947

Epoch 16/30

3/3 [==============================] - 0s 26ms/step - loss: 0.3217 - accuracy: 0.9583 - val\_loss: 0.3785 - val\_accuracy: 0.8421

Epoch 17/30

3/3 [==============================] - 0s 26ms/step - loss: 0.3050 - accuracy: 0.9702 - val\_loss: 0.3609 - val\_accuracy: 0.8947

Epoch 18/30

3/3 [==============================] - 0s 25ms/step - loss: 0.2854 - accuracy: 0.9643 - val\_loss: 0.3349 - val\_accuracy: 0.8947

Epoch 19/30

3/3 [==============================] - 0s 26ms/step - loss: 0.2711 - accuracy: 0.9613 - val\_loss: 0.3233 - val\_accuracy: 0.8947

Epoch 20/30

3/3 [==============================] - 0s 25ms/step - loss: 0.2556 - accuracy: 0.9702 - val\_loss: 0.3288 - val\_accuracy: 0.8947

Epoch 21/30

3/3 [==============================] - 0s 26ms/step - loss: 0.2432 - accuracy: 0.9732 - val\_loss: 0.3256 - val\_accuracy: 0.8947

Epoch 22/30

3/3 [==============================] - 0s 25ms/step - loss: 0.2313 - accuracy: 0.9732 - val\_loss: 0.3038 - val\_accuracy: 0.8947

Epoch 23/30

3/3 [==============================] - 0s 26ms/step - loss: 0.2187 - accuracy: 0.9702 - val\_loss: 0.3032 - val\_accuracy: 0.8947

Epoch 24/30

3/3 [==============================] - 0s 26ms/step - loss: 0.2079 - accuracy: 0.9792 - val\_loss: 0.2917 - val\_accuracy: 0.9211

Epoch 25/30

3/3 [==============================] - 0s 26ms/step - loss: 0.1970 - accuracy: 0.9851 - val\_loss: 0.2833 - val\_accuracy: 0.8947

Epoch 26/30

3/3 [==============================] - 0s 26ms/step - loss: 0.1880 - accuracy: 0.9792 - val\_loss: 0.2820 - val\_accuracy: 0.8947

Epoch 27/30

3/3 [==============================] - 0s 25ms/step - loss: 0.1786 - accuracy: 0.9792 - val\_loss: 0.2808 - val\_accuracy: 0.8947

Epoch 28/30

3/3 [==============================] - 0s 25ms/step - loss: 0.1714 - accuracy: 0.9792 - val\_loss: 0.2705 - val\_accuracy: 0.9211

Epoch 29/30

3/3 [==============================] - 0s 24ms/step - loss: 0.1619 - accuracy: 0.9851 - val\_loss: 0.2545 - val\_accuracy: 0.8947

Epoch 30/30

3/3 [==============================] - 0s 25ms/step - loss: 0.1561 - accuracy: 0.9762 - val\_loss: 0.2541 - val\_accuracy: 0.8947

Epoch 1/30

3/3 [==============================] - 9s 4s/step - loss: 1.9457 - accuracy: 0.3185 - val\_loss: 1.1120 - val\_accuracy: 0.4474

Epoch 2/30

3/3 [==============================] - 0s 27ms/step - loss: 1.1743 - accuracy: 0.4494 - val\_loss: 0.9360 - val\_accuracy: 0.5263

Epoch 3/30

3/3 [==============================] - 0s 39ms/step - loss: 0.7434 - accuracy: 0.6548 - val\_loss: 0.7346 - val\_accuracy: 0.6316

Epoch 4/30

3/3 [==============================] - 0s 27ms/step - loss: 0.7310 - accuracy: 0.5863 - val\_loss: 0.6548 - val\_accuracy: 0.6579

Epoch 5/30

3/3 [==============================] - 0s 32ms/step - loss: 0.5905 - accuracy: 0.7679 - val\_loss: 0.6854 - val\_accuracy: 0.7632

Epoch 6/30

3/3 [==============================] - 0s 76ms/step - loss: 0.5524 - accuracy: 0.8006 - val\_loss: 0.6915 - val\_accuracy: 0.7368

Epoch 7/30

3/3 [==============================] - 0s 26ms/step - loss: 0.4984 - accuracy: 0.8333 - val\_loss: 0.5709 - val\_accuracy: 0.7895

Epoch 8/30

3/3 [==============================] - 0s 26ms/step - loss: 0.4397 - accuracy: 0.8601 - val\_loss: 0.5289 - val\_accuracy: 0.7895

Epoch 9/30

3/3 [==============================] - 0s 94ms/step - loss: 0.4147 - accuracy: 0.8839 - val\_loss: 0.5075 - val\_accuracy: 0.7895

Epoch 10/30

3/3 [==============================] - 0s 26ms/step - loss: 0.3649 - accuracy: 0.8839 - val\_loss: 0.5025 - val\_accuracy: 0.8158

Epoch 11/30

3/3 [==============================] - 0s 29ms/step - loss: 0.3455 - accuracy: 0.9018 - val\_loss: 0.4856 - val\_accuracy: 0.8158

Epoch 12/30

3/3 [==============================] - 0s 26ms/step - loss: 0.3185 - accuracy: 0.9107 - val\_loss: 0.4475 - val\_accuracy: 0.8421

Epoch 13/30

3/3 [==============================] - 0s 27ms/step - loss: 0.2908 - accuracy: 0.9375 - val\_loss: 0.4079 - val\_accuracy: 0.8421

Epoch 14/30

3/3 [==============================] - 0s 77ms/step - loss: 0.2730 - accuracy: 0.9554 - val\_loss: 0.3725 - val\_accuracy: 0.8421

Epoch 15/30

3/3 [==============================] - 0s 26ms/step - loss: 0.2556 - accuracy: 0.9613 - val\_loss: 0.3761 - val\_accuracy: 0.8421

Epoch 16/30

3/3 [==============================] - 0s 26ms/step - loss: 0.2433 - accuracy: 0.9494 - val\_loss: 0.3806 - val\_accuracy: 0.8421

Epoch 17/30

3/3 [==============================] - 0s 115ms/step - loss: 0.2249 - accuracy: 0.9613 - val\_loss: 0.3207 - val\_accuracy: 0.8947

Epoch 18/30

3/3 [==============================] - 0s 27ms/step - loss: 0.2140 - accuracy: 0.9673 - val\_loss: 0.3164 - val\_accuracy: 0.8947

Epoch 19/30

3/3 [==============================] - 0s 27ms/step - loss: 0.1969 - accuracy: 0.9702 - val\_loss: 0.3268 - val\_accuracy: 0.8684

Epoch 20/30

3/3 [==============================] - 0s 26ms/step - loss: 0.1869 - accuracy: 0.9673 - val\_loss: 0.3099 - val\_accuracy: 0.8684

Epoch 21/30

3/3 [==============================] - 0s 26ms/step - loss: 0.1776 - accuracy: 0.9673 - val\_loss: 0.2882 - val\_accuracy: 0.8684

Epoch 22/30

3/3 [==============================] - 0s 28ms/step - loss: 0.1648 - accuracy: 0.9762 - val\_loss: 0.2680 - val\_accuracy: 0.9211

Epoch 23/30

3/3 [==============================] - 0s 27ms/step - loss: 0.1559 - accuracy: 0.9792 - val\_loss: 0.2835 - val\_accuracy: 0.9211

Epoch 24/30

3/3 [==============================] - 0s 27ms/step - loss: 0.1501 - accuracy: 0.9792 - val\_loss: 0.2721 - val\_accuracy: 0.8684

Epoch 25/30

3/3 [==============================] - 0s 26ms/step - loss: 0.1379 - accuracy: 0.9851 - val\_loss: 0.2366 - val\_accuracy: 0.9211

Epoch 26/30

3/3 [==============================] - 0s 26ms/step - loss: 0.1314 - accuracy: 0.9851 - val\_loss: 0.2475 - val\_accuracy: 0.8947

Epoch 27/30

3/3 [==============================] - 0s 26ms/step - loss: 0.1218 - accuracy: 0.9821 - val\_loss: 0.2529 - val\_accuracy: 0.8947

Epoch 28/30

3/3 [==============================] - 0s 25ms/step - loss: 0.1165 - accuracy: 0.9851 - val\_loss: 0.2322 - val\_accuracy: 0.9211

Epoch 29/30

3/3 [==============================] - 0s 26ms/step - loss: 0.1088 - accuracy: 0.9881 - val\_loss: 0.2127 - val\_accuracy: 0.9474

Epoch 30/30

3/3 [==============================] - 0s 25ms/step - loss: 0.1040 - accuracy: 0.9911 - val\_loss: 0.2107 - val\_accuracy: 0.9474

Epoch 1/30

3/3 [==============================] - 1s 474ms/step - loss: 1.7749 - accuracy: 0.3720 - val\_loss: 1.3461 - val\_accuracy: 0.2895

Epoch 2/30

3/3 [==============================] - 0s 27ms/step - loss: 1.2835 - accuracy: 0.3006 - val\_loss: 1.1429 - val\_accuracy: 0.3421

Epoch 3/30

3/3 [==============================] - 0s 26ms/step - loss: 1.0178 - accuracy: 0.4077 - val\_loss: 0.9661 - val\_accuracy: 0.5263

Epoch 4/30

3/3 [==============================] - 0s 27ms/step - loss: 0.9311 - accuracy: 0.4583 - val\_loss: 0.9337 - val\_accuracy: 0.5263

Epoch 5/30

3/3 [==============================] - 0s 27ms/step - loss: 0.8390 - accuracy: 0.6369 - val\_loss: 0.8145 - val\_accuracy: 0.7105

Epoch 6/30

3/3 [==============================] - 0s 42ms/step - loss: 0.7548 - accuracy: 0.7560 - val\_loss: 0.7838 - val\_accuracy: 0.7632

Epoch 7/30

3/3 [==============================] - 0s 42ms/step - loss: 0.6722 - accuracy: 0.7619 - val\_loss: 0.7272 - val\_accuracy: 0.7368

Epoch 8/30

3/3 [==============================] - 0s 43ms/step - loss: 0.5931 - accuracy: 0.8125 - val\_loss: 0.7084 - val\_accuracy: 0.7105

Epoch 9/30

3/3 [==============================] - 0s 42ms/step - loss: 0.5552 - accuracy: 0.7946 - val\_loss: 0.6677 - val\_accuracy: 0.7368

Epoch 10/30

3/3 [==============================] - 0s 42ms/step - loss: 0.5147 - accuracy: 0.8214 - val\_loss: 0.6298 - val\_accuracy: 0.7368

Epoch 11/30

3/3 [==============================] - 0s 26ms/step - loss: 0.4676 - accuracy: 0.8452 - val\_loss: 0.6174 - val\_accuracy: 0.7632

Epoch 12/30

3/3 [==============================] - 0s 26ms/step - loss: 0.4477 - accuracy: 0.8512 - val\_loss: 0.6207 - val\_accuracy: 0.7368

Epoch 13/30

3/3 [==============================] - 0s 42ms/step - loss: 0.4168 - accuracy: 0.8780 - val\_loss: 0.5873 - val\_accuracy: 0.7632

Epoch 14/30

3/3 [==============================] - 0s 42ms/step - loss: 0.3890 - accuracy: 0.9048 - val\_loss: 0.5802 - val\_accuracy: 0.7368

Epoch 15/30

3/3 [==============================] - 0s 42ms/step - loss: 0.3655 - accuracy: 0.9226 - val\_loss: 0.5685 - val\_accuracy: 0.7632

Epoch 16/30

3/3 [==============================] - 0s 26ms/step - loss: 0.3427 - accuracy: 0.9137 - val\_loss: 0.5572 - val\_accuracy: 0.7895

Epoch 17/30

3/3 [==============================] - 0s 43ms/step - loss: 0.3242 - accuracy: 0.9226 - val\_loss: 0.5403 - val\_accuracy: 0.7895

Epoch 18/30

3/3 [==============================] - 0s 27ms/step - loss: 0.3052 - accuracy: 0.9494 - val\_loss: 0.5318 - val\_accuracy: 0.7895

Epoch 19/30

3/3 [==============================] - 0s 27ms/step - loss: 0.2869 - accuracy: 0.9554 - val\_loss: 0.5300 - val\_accuracy: 0.7895

Epoch 20/30

3/3 [==============================] - 0s 27ms/step - loss: 0.2758 - accuracy: 0.9435 - val\_loss: 0.5351 - val\_accuracy: 0.7895

Epoch 21/30

3/3 [==============================] - 0s 27ms/step - loss: 0.2581 - accuracy: 0.9494 - val\_loss: 0.5080 - val\_accuracy: 0.7895

Epoch 22/30

3/3 [==============================] - 0s 27ms/step - loss: 0.2460 - accuracy: 0.9583 - val\_loss: 0.5009 - val\_accuracy: 0.7895

Epoch 23/30

3/3 [==============================] - 0s 27ms/step - loss: 0.2345 - accuracy: 0.9613 - val\_loss: 0.5130 - val\_accuracy: 0.7895

Epoch 24/30

3/3 [==============================] - 0s 26ms/step - loss: 0.2186 - accuracy: 0.9613 - val\_loss: 0.4981 - val\_accuracy: 0.7895

Epoch 25/30

3/3 [==============================] - 0s 26ms/step - loss: 0.2066 - accuracy: 0.9702 - val\_loss: 0.4884 - val\_accuracy: 0.7895

Epoch 26/30

3/3 [==============================] - 0s 26ms/step - loss: 0.1973 - accuracy: 0.9732 - val\_loss: 0.4837 - val\_accuracy: 0.7895

Epoch 27/30

3/3 [==============================] - 0s 27ms/step - loss: 0.1863 - accuracy: 0.9762 - val\_loss: 0.4889 - val\_accuracy: 0.7895

Epoch 28/30

3/3 [==============================] - 0s 26ms/step - loss: 0.1775 - accuracy: 0.9702 - val\_loss: 0.4861 - val\_accuracy: 0.7895

Epoch 29/30

3/3 [==============================] - 0s 26ms/step - loss: 0.1695 - accuracy: 0.9762 - val\_loss: 0.4721 - val\_accuracy: 0.7895

Epoch 30/30

3/3 [==============================] - 0s 45ms/step - loss: 0.1603 - accuracy: 0.9792 - val\_loss: 0.4729 - val\_accuracy: 0.7895

Epoch 1/30

3/3 [==============================] - 1s 154ms/step - loss: 2.7510 - accuracy: 0.3363 - val\_loss: 1.5319 - val\_accuracy: 0.4211

Epoch 2/30

3/3 [==============================] - 0s 27ms/step - loss: 1.1980 - accuracy: 0.4375 - val\_loss: 1.1206 - val\_accuracy: 0.5263

Epoch 3/30

3/3 [==============================] - 0s 26ms/step - loss: 1.1504 - accuracy: 0.3869 - val\_loss: 0.9772 - val\_accuracy: 0.5789

Epoch 4/30

3/3 [==============================] - 0s 28ms/step - loss: 0.9401 - accuracy: 0.5923 - val\_loss: 0.9359 - val\_accuracy: 0.6316

Epoch 5/30

3/3 [==============================] - 0s 26ms/step - loss: 0.8436 - accuracy: 0.6845 - val\_loss: 0.9847 - val\_accuracy: 0.6316

Epoch 6/30

3/3 [==============================] - 0s 27ms/step - loss: 0.8304 - accuracy: 0.6726 - val\_loss: 0.9666 - val\_accuracy: 0.5263

Epoch 7/30

3/3 [==============================] - 0s 27ms/step - loss: 0.7829 - accuracy: 0.6964 - val\_loss: 0.8624 - val\_accuracy: 0.7105

Epoch 8/30

3/3 [==============================] - 0s 26ms/step - loss: 0.7060 - accuracy: 0.7857 - val\_loss: 0.7728 - val\_accuracy: 0.7632

Epoch 9/30

3/3 [==============================] - 0s 27ms/step - loss: 0.6434 - accuracy: 0.8065 - val\_loss: 0.7108 - val\_accuracy: 0.8421

Epoch 10/30

3/3 [==============================] - 0s 27ms/step - loss: 0.5964 - accuracy: 0.8244 - val\_loss: 0.6741 - val\_accuracy: 0.8421

Epoch 11/30

3/3 [==============================] - 0s 27ms/step - loss: 0.5559 - accuracy: 0.8482 - val\_loss: 0.6590 - val\_accuracy: 0.8421

Epoch 12/30

3/3 [==============================] - 0s 27ms/step - loss: 0.5224 - accuracy: 0.8512 - val\_loss: 0.6406 - val\_accuracy: 0.8684

Epoch 13/30

3/3 [==============================] - 0s 26ms/step - loss: 0.4932 - accuracy: 0.8631 - val\_loss: 0.6058 - val\_accuracy: 0.8684

Epoch 14/30

3/3 [==============================] - 0s 27ms/step - loss: 0.4660 - accuracy: 0.8780 - val\_loss: 0.5742 - val\_accuracy: 0.8684

Epoch 15/30

3/3 [==============================] - 0s 26ms/step - loss: 0.4425 - accuracy: 0.8958 - val\_loss: 0.5472 - val\_accuracy: 0.8684

Epoch 16/30

3/3 [==============================] - 0s 26ms/step - loss: 0.4193 - accuracy: 0.8929 - val\_loss: 0.5194 - val\_accuracy: 0.8684

Epoch 17/30

3/3 [==============================] - 0s 27ms/step - loss: 0.3999 - accuracy: 0.8958 - val\_loss: 0.4990 - val\_accuracy: 0.8684

Epoch 18/30

3/3 [==============================] - 0s 27ms/step - loss: 0.3800 - accuracy: 0.9107 - val\_loss: 0.4818 - val\_accuracy: 0.9211

Epoch 19/30

3/3 [==============================] - 0s 27ms/step - loss: 0.3604 - accuracy: 0.9107 - val\_loss: 0.4644 - val\_accuracy: 0.8947

Epoch 20/30

3/3 [==============================] - 0s 26ms/step - loss: 0.3436 - accuracy: 0.9137 - val\_loss: 0.4495 - val\_accuracy: 0.8947

Epoch 21/30

3/3 [==============================] - 0s 27ms/step - loss: 0.3271 - accuracy: 0.9226 - val\_loss: 0.4269 - val\_accuracy: 0.9211

Epoch 22/30

3/3 [==============================] - 0s 27ms/step - loss: 0.3115 - accuracy: 0.9226 - val\_loss: 0.4102 - val\_accuracy: 0.9474

Epoch 23/30

3/3 [==============================] - 0s 26ms/step - loss: 0.2973 - accuracy: 0.9405 - val\_loss: 0.3901 - val\_accuracy: 0.9474

Epoch 24/30

3/3 [==============================] - 0s 27ms/step - loss: 0.2837 - accuracy: 0.9583 - val\_loss: 0.3790 - val\_accuracy: 0.9474

Epoch 25/30

3/3 [==============================] - 0s 27ms/step - loss: 0.2698 - accuracy: 0.9613 - val\_loss: 0.3691 - val\_accuracy: 0.9474

Epoch 26/30

3/3 [==============================] - 0s 26ms/step - loss: 0.2585 - accuracy: 0.9643 - val\_loss: 0.3539 - val\_accuracy: 0.9474

Epoch 27/30

3/3 [==============================] - 0s 26ms/step - loss: 0.2455 - accuracy: 0.9643 - val\_loss: 0.3490 - val\_accuracy: 0.9474

Epoch 28/30

3/3 [==============================] - 0s 26ms/step - loss: 0.2350 - accuracy: 0.9762 - val\_loss: 0.3405 - val\_accuracy: 0.9474

Epoch 29/30

3/3 [==============================] - 0s 25ms/step - loss: 0.2247 - accuracy: 0.9792 - val\_loss: 0.3298 - val\_accuracy: 0.9474

Epoch 30/30

3/3 [==============================] - 0s 26ms/step - loss: 0.2157 - accuracy: 0.9732 - val\_loss: 0.3241 - val\_accuracy: 0.9474

Epoch 1/30

3/3 [==============================] - 1s 147ms/step - loss: 1.8344 - accuracy: 0.3839 - val\_loss: 1.4650 - val\_accuracy: 0.1579

Epoch 2/30

3/3 [==============================] - 0s 41ms/step - loss: 1.1426 - accuracy: 0.3839 - val\_loss: 1.1806 - val\_accuracy: 0.3158

Epoch 3/30

3/3 [==============================] - 0s 26ms/step - loss: 1.0363 - accuracy: 0.3720 - val\_loss: 0.9894 - val\_accuracy: 0.3158

Epoch 4/30

3/3 [==============================] - 0s 25ms/step - loss: 0.9120 - accuracy: 0.5744 - val\_loss: 0.8860 - val\_accuracy: 0.6842

Epoch 5/30

3/3 [==============================] - 0s 25ms/step - loss: 0.8433 - accuracy: 0.6220 - val\_loss: 0.9094 - val\_accuracy: 0.5526

Epoch 6/30

3/3 [==============================] - 0s 41ms/step - loss: 0.8208 - accuracy: 0.6548 - val\_loss: 0.8905 - val\_accuracy: 0.6316

Epoch 7/30

3/3 [==============================] - 0s 41ms/step - loss: 0.7548 - accuracy: 0.7262 - val\_loss: 0.8085 - val\_accuracy: 0.6842

Epoch 8/30

3/3 [==============================] - 0s 40ms/step - loss: 0.6706 - accuracy: 0.7946 - val\_loss: 0.7757 - val\_accuracy: 0.6842

Epoch 9/30

3/3 [==============================] - 0s 40ms/step - loss: 0.6414 - accuracy: 0.7768 - val\_loss: 0.7657 - val\_accuracy: 0.6579

Epoch 10/30

3/3 [==============================] - 0s 41ms/step - loss: 0.6036 - accuracy: 0.7679 - val\_loss: 0.7092 - val\_accuracy: 0.7368

Epoch 11/30

3/3 [==============================] - 0s 25ms/step - loss: 0.5470 - accuracy: 0.8363 - val\_loss: 0.6631 - val\_accuracy: 0.7368

Epoch 12/30

3/3 [==============================] - 0s 24ms/step - loss: 0.5092 - accuracy: 0.8601 - val\_loss: 0.6230 - val\_accuracy: 0.7895

Epoch 13/30

3/3 [==============================] - 0s 41ms/step - loss: 0.4727 - accuracy: 0.8631 - val\_loss: 0.5931 - val\_accuracy: 0.7895

Epoch 14/30

3/3 [==============================] - 0s 41ms/step - loss: 0.4395 - accuracy: 0.8661 - val\_loss: 0.5826 - val\_accuracy: 0.7895

Epoch 15/30

3/3 [==============================] - 0s 41ms/step - loss: 0.4225 - accuracy: 0.8929 - val\_loss: 0.5540 - val\_accuracy: 0.8158

Epoch 16/30

3/3 [==============================] - 0s 41ms/step - loss: 0.3968 - accuracy: 0.9048 - val\_loss: 0.5167 - val\_accuracy: 0.8684

Epoch 17/30

3/3 [==============================] - 0s 25ms/step - loss: 0.3710 - accuracy: 0.9107 - val\_loss: 0.4972 - val\_accuracy: 0.8684

Epoch 18/30

3/3 [==============================] - 0s 28ms/step - loss: 0.3476 - accuracy: 0.9226 - val\_loss: 0.4735 - val\_accuracy: 0.8947

Epoch 19/30

3/3 [==============================] - 0s 25ms/step - loss: 0.3267 - accuracy: 0.9345 - val\_loss: 0.4472 - val\_accuracy: 0.8947

Epoch 20/30

3/3 [==============================] - 0s 25ms/step - loss: 0.3074 - accuracy: 0.9315 - val\_loss: 0.4237 - val\_accuracy: 0.8947

Epoch 21/30

3/3 [==============================] - 0s 25ms/step - loss: 0.2906 - accuracy: 0.9435 - val\_loss: 0.4046 - val\_accuracy: 0.8947

Epoch 22/30

3/3 [==============================] - 0s 25ms/step - loss: 0.2733 - accuracy: 0.9435 - val\_loss: 0.3849 - val\_accuracy: 0.9211

Epoch 23/30

3/3 [==============================] - 0s 24ms/step - loss: 0.2573 - accuracy: 0.9524 - val\_loss: 0.3672 - val\_accuracy: 0.9211

Epoch 24/30

3/3 [==============================] - 0s 41ms/step - loss: 0.2432 - accuracy: 0.9554 - val\_loss: 0.3479 - val\_accuracy: 0.9211

Epoch 25/30

3/3 [==============================] - 0s 42ms/step - loss: 0.2299 - accuracy: 0.9554 - val\_loss: 0.3305 - val\_accuracy: 0.9211

Epoch 26/30

3/3 [==============================] - 0s 25ms/step - loss: 0.2155 - accuracy: 0.9643 - val\_loss: 0.3167 - val\_accuracy: 0.9211

Epoch 27/30

3/3 [==============================] - 0s 25ms/step - loss: 0.2045 - accuracy: 0.9643 - val\_loss: 0.3018 - val\_accuracy: 0.9211

Epoch 28/30

3/3 [==============================] - 0s 41ms/step - loss: 0.1927 - accuracy: 0.9643 - val\_loss: 0.2874 - val\_accuracy: 0.9211

Epoch 29/30

3/3 [==============================] - 0s 25ms/step - loss: 0.1805 - accuracy: 0.9702 - val\_loss: 0.2753 - val\_accuracy: 0.9211

Epoch 30/30

3/3 [==============================] - 0s 25ms/step - loss: 0.1714 - accuracy: 0.9702 - val\_loss: 0.2620 - val\_accuracy: 0.9211

Epoch 1/30

3/3 [==============================] - 1s 148ms/step - loss: 1.6787 - accuracy: 0.3601 - val\_loss: 0.7429 - val\_accuracy: 0.6842

Epoch 2/30

3/3 [==============================] - 0s 31ms/step - loss: 1.0083 - accuracy: 0.4732 - val\_loss: 0.6197 - val\_accuracy: 0.7105

Epoch 3/30

3/3 [==============================] - 0s 25ms/step - loss: 0.7387 - accuracy: 0.7113 - val\_loss: 0.8185 - val\_accuracy: 0.6053

Epoch 4/30

3/3 [==============================] - 0s 26ms/step - loss: 0.6531 - accuracy: 0.6786 - val\_loss: 0.4629 - val\_accuracy: 0.8421

Epoch 5/30

3/3 [==============================] - 0s 27ms/step - loss: 0.5214 - accuracy: 0.8036 - val\_loss: 0.4312 - val\_accuracy: 0.7895

Epoch 6/30

3/3 [==============================] - 0s 27ms/step - loss: 0.4630 - accuracy: 0.8185 - val\_loss: 0.4339 - val\_accuracy: 0.8158

Epoch 7/30

3/3 [==============================] - 0s 26ms/step - loss: 0.3926 - accuracy: 0.8542 - val\_loss: 0.5172 - val\_accuracy: 0.7368

Epoch 8/30

3/3 [==============================] - 0s 27ms/step - loss: 0.3632 - accuracy: 0.8690 - val\_loss: 0.3695 - val\_accuracy: 0.8684

Epoch 9/30

3/3 [==============================] - 0s 26ms/step - loss: 0.2982 - accuracy: 0.9226 - val\_loss: 0.3210 - val\_accuracy: 0.8947

Epoch 10/30

3/3 [==============================] - 0s 26ms/step - loss: 0.2966 - accuracy: 0.9226 - val\_loss: 0.3309 - val\_accuracy: 0.8684

Epoch 11/30

3/3 [==============================] - 0s 26ms/step - loss: 0.2474 - accuracy: 0.9375 - val\_loss: 0.3854 - val\_accuracy: 0.8421

Epoch 12/30

3/3 [==============================] - 0s 27ms/step - loss: 0.2381 - accuracy: 0.9375 - val\_loss: 0.3199 - val\_accuracy: 0.8684

Epoch 13/30

3/3 [==============================] - 0s 26ms/step - loss: 0.2223 - accuracy: 0.9375 - val\_loss: 0.2571 - val\_accuracy: 0.9211

Epoch 14/30

3/3 [==============================] - 0s 26ms/step - loss: 0.2056 - accuracy: 0.9435 - val\_loss: 0.2851 - val\_accuracy: 0.8684

Epoch 15/30

3/3 [==============================] - 0s 26ms/step - loss: 0.1931 - accuracy: 0.9524 - val\_loss: 0.3167 - val\_accuracy: 0.8684

Epoch 16/30

3/3 [==============================] - 0s 26ms/step - loss: 0.1762 - accuracy: 0.9732 - val\_loss: 0.2494 - val\_accuracy: 0.8947

Epoch 17/30

3/3 [==============================] - 0s 26ms/step - loss: 0.1647 - accuracy: 0.9643 - val\_loss: 0.2249 - val\_accuracy: 0.9211

Epoch 18/30

3/3 [==============================] - 0s 32ms/step - loss: 0.1578 - accuracy: 0.9613 - val\_loss: 0.2351 - val\_accuracy: 0.8947

Epoch 19/30

3/3 [==============================] - 0s 26ms/step - loss: 0.1458 - accuracy: 0.9732 - val\_loss: 0.2667 - val\_accuracy: 0.8684

Epoch 20/30

3/3 [==============================] - 0s 26ms/step - loss: 0.1422 - accuracy: 0.9643 - val\_loss: 0.2336 - val\_accuracy: 0.9211

Epoch 21/30

3/3 [==============================] - 0s 26ms/step - loss: 0.1326 - accuracy: 0.9792 - val\_loss: 0.2036 - val\_accuracy: 0.9211

Epoch 22/30

3/3 [==============================] - 0s 25ms/step - loss: 0.1265 - accuracy: 0.9792 - val\_loss: 0.2081 - val\_accuracy: 0.9211

Epoch 23/30

3/3 [==============================] - 0s 26ms/step - loss: 0.1190 - accuracy: 0.9792 - val\_loss: 0.2004 - val\_accuracy: 0.9211

Epoch 24/30

3/3 [==============================] - 0s 26ms/step - loss: 0.1132 - accuracy: 0.9792 - val\_loss: 0.1918 - val\_accuracy: 0.9211

Epoch 25/30

3/3 [==============================] - 0s 26ms/step - loss: 0.1087 - accuracy: 0.9792 - val\_loss: 0.1825 - val\_accuracy: 0.9474

Epoch 26/30

3/3 [==============================] - 0s 26ms/step - loss: 0.1040 - accuracy: 0.9821 - val\_loss: 0.1832 - val\_accuracy: 0.9474

Epoch 27/30

3/3 [==============================] - 0s 26ms/step - loss: 0.0994 - accuracy: 0.9821 - val\_loss: 0.1760 - val\_accuracy: 0.9211

Epoch 28/30

3/3 [==============================] - 0s 26ms/step - loss: 0.0953 - accuracy: 0.9821 - val\_loss: 0.1701 - val\_accuracy: 0.9211

Epoch 29/30

3/3 [==============================] - 0s 25ms/step - loss: 0.0909 - accuracy: 0.9821 - val\_loss: 0.1632 - val\_accuracy: 0.9474

Epoch 30/30

3/3 [==============================] - 0s 25ms/step - loss: 0.0875 - accuracy: 0.9821 - val\_loss: 0.1545 - val\_accuracy: 0.9737

Accuracy : 0.9372549019607843

15/15 [==============================] - 38s 2s/step

8/8 [==============================] - 18s 2s/step

Model: "model\_7"

\_\_\_\_\_\_\_\_\_\_\_\_\_\_\_\_\_\_\_\_\_\_\_\_\_\_\_\_\_\_\_\_\_\_\_\_\_\_\_\_\_\_\_\_\_\_\_\_\_\_\_\_\_\_\_\_\_\_\_\_\_\_\_\_\_

Layer (type) Output Shape Param #

=================================================================

input\_16 (InputLayer) [(None, 9, 9, 512)] 0

  

block5\_conv2 (Conv2D) multiple 2359808

  

block5\_conv3 (Conv2D) multiple 2359808

  

block5\_conv4 (Conv2D) multiple 2359808

  

block5\_pool (MaxPooling2D) multiple 0

  

conv2d\_15 (Conv2D) (None, 2, 2, 64) 294976

  

max\_pooling2d\_15 (MaxPoolin (None, 1, 1, 64) 0

g2D)

  

flatten\_53 (Flatten) (None, 64) 0

  

dense\_106 (Dense) (None, 100) 6500

  

dense\_107 (Dense) (None, 3) 303

  

=================================================================

Total params: 7,381,203

Trainable params: 7,381,203

Non-trainable params: 0

\_\_\_\_\_\_\_\_\_\_\_\_\_\_\_\_\_\_\_\_\_\_\_\_\_\_\_\_\_\_\_\_\_\_\_\_\_\_\_\_\_\_\_\_\_\_\_\_\_\_\_\_\_\_\_\_\_\_\_\_\_\_\_\_\_

Epoch 1/30

3/3 [==============================] - 10s 2s/step - loss: 1.5667 - acc: 0.4332 - val\_loss: 1.0432 - val\_acc: 0.3617

Epoch 2/30

3/3 [==============================] - 8s 3s/step - loss: 1.0411 - acc: 0.4332 - val\_loss: 0.8309 - val\_acc: 0.6383

Epoch 3/30

3/3 [==============================] - 7s 2s/step - loss: 1.2502 - acc: 0.4492 - val\_loss: 1.0121 - val\_acc: 0.4149

Epoch 4/30

3/3 [==============================] - 8s 3s/step - loss: 1.0394 - acc: 0.5160 - val\_loss: 0.8572 - val\_acc: 0.7234

Epoch 5/30

3/3 [==============================] - 7s 2s/step - loss: 0.8673 - acc: 0.6497 - val\_loss: 0.7402 - val\_acc: 0.6702

Epoch 6/30

3/3 [==============================] - 7s 2s/step - loss: 0.7957 - acc: 0.6524 - val\_loss: 0.5703 - val\_acc: 0.7660

Epoch 7/30

3/3 [==============================] - 7s 2s/step - loss: 0.6484 - acc: 0.7299 - val\_loss: 0.5787 - val\_acc: 0.7979

Epoch 8/30

3/3 [==============================] - 7s 2s/step - loss: 0.5631 - acc: 0.7433 - val\_loss: 0.4092 - val\_acc: 0.8404

Epoch 9/30

3/3 [==============================] - 7s 2s/step - loss: 0.5538 - acc: 0.7540 - val\_loss: 0.4800 - val\_acc: 0.7872

Epoch 10/30

3/3 [==============================] - 7s 2s/step - loss: 0.4476 - acc: 0.7968 - val\_loss: 0.3790 - val\_acc: 0.8830

Epoch 11/30

3/3 [==============================] - 7s 2s/step - loss: 0.3605 - acc: 0.8503 - val\_loss: 0.4308 - val\_acc: 0.8085

Epoch 12/30

3/3 [==============================] - 7s 3s/step - loss: 0.3429 - acc: 0.8529 - val\_loss: 0.3909 - val\_acc: 0.8936

Epoch 13/30

3/3 [==============================] - 7s 2s/step - loss: 0.2368 - acc: 0.9171 - val\_loss: 0.4492 - val\_acc: 0.8723

Epoch 14/30

3/3 [==============================] - 7s 2s/step - loss: 0.1964 - acc: 0.9332 - val\_loss: 0.3636 - val\_acc: 0.8936

Epoch 15/30

3/3 [==============================] - 7s 2s/step - loss: 0.1515 - acc: 0.9385 - val\_loss: 0.4109 - val\_acc: 0.8936

Epoch 16/30

3/3 [==============================] - 7s 2s/step - loss: 0.1578 - acc: 0.9545 - val\_loss: 0.5309 - val\_acc: 0.8617

Epoch 17/30

3/3 [==============================] - 7s 2s/step - loss: 0.1421 - acc: 0.9439 - val\_loss: 0.4405 - val\_acc: 0.9149

Epoch 18/30

3/3 [==============================] - 7s 2s/step - loss: 0.1177 - acc: 0.9572 - val\_loss: 0.5223 - val\_acc: 0.9043

Epoch 19/30

3/3 [==============================] - 7s 2s/step - loss: 0.0709 - acc: 0.9759 - val\_loss: 0.4388 - val\_acc: 0.8936

Epoch 20/30

3/3 [==============================] - 7s 2s/step - loss: 0.0564 - acc: 0.9813 - val\_loss: 0.6611 - val\_acc: 0.8936

Epoch 21/30

3/3 [==============================] - 7s 2s/step - loss: 0.0559 - acc: 0.9840 - val\_loss: 0.5682 - val\_acc: 0.9149

Epoch 22/30

3/3 [==============================] - 7s 2s/step - loss: 0.0524 - acc: 0.9920 - val\_loss: 0.6598 - val\_acc: 0.9149

Epoch 23/30

3/3 [==============================] - 7s 2s/step - loss: 0.0169 - acc: 0.9947 - val\_loss: 0.7222 - val\_acc: 0.9043

Epoch 24/30

3/3 [==============================] - 7s 2s/step - loss: 0.0245 - acc: 0.9947 - val\_loss: 0.7224 - val\_acc: 0.9149

Epoch 25/30

3/3 [==============================] - 7s 2s/step - loss: 0.0233 - acc: 0.9947 - val\_loss: 0.5415 - val\_acc: 0.9043

Epoch 26/30

3/3 [==============================] - 7s 2s/step - loss: 0.0422 - acc: 0.9866 - val\_loss: 0.7672 - val\_acc: 0.9149

Epoch 27/30

3/3 [==============================] - 7s 2s/step - loss: 0.0098 - acc: 1.0000 - val\_loss: 0.7243 - val\_acc: 0.9149

Epoch 28/30

3/3 [==============================] - 7s 2s/step - loss: 0.0123 - acc: 0.9973 - val\_loss: 0.6559 - val\_acc: 0.9468

Epoch 29/30

3/3 [==============================] - 7s 2s/step - loss: 0.0061 - acc: 1.0000 - val\_loss: 0.7002 - val\_acc: 0.9149

Epoch 30/30

3/3 [==============================] - 7s 2s/step - loss: 0.0052 - acc: 1.0000 - val\_loss: 0.6893 - val\_acc: 0.9362

VGG19 Fine Tuning Accuracy :

  

Accuracy : 0.9372549019607843

8/8 [==============================] - 2s 222ms/step - loss: 0.1721 - acc: 0.9843

\_\_\_\_\_\_\_\_\_\_\_\_\_\_\_\_\_\_\_\_\_\_\_\_\_\_\_\_\_\_\_\_\_\_

VGG16 Fine Tuning" :

\_\_\_\_\_\_\_\_\_\_\_\_\_\_\_\_\_\_\_\_\_\_\_\_\_\_\_\_\_\_\_\_\_\_\_\_\_

Model MAE MSE RMSE R2 Square Accuracy

0 VGG19 Fine Tuning 0.086275 0.133333 0.365148 0.797742 0.937255

In [10]:
