# Supplementary figures and images for: Classification of melanonychia, Beau’s lines, and nail clubbing based on nail images and transfer learning techniques
Source: PeerJ Comput Sci. 2023 Aug 24;9:e1533. doi: 10.7717/peerj-cs.1533 (PMC10495933; doi:10.7717/peerj-cs.1533)

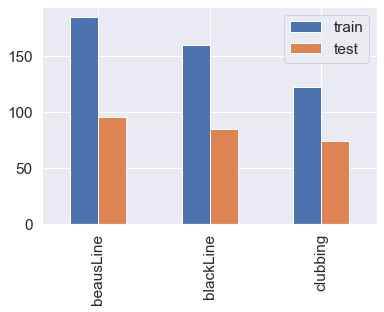

Supplement: Supplemental Information 1 [file peerj-cs-09-1533-s001.zip › Codes and Console Outputs Supplementary Files/Code and Console Outputs for VGG16/Figure 2023-03-09 143103 (0).png]

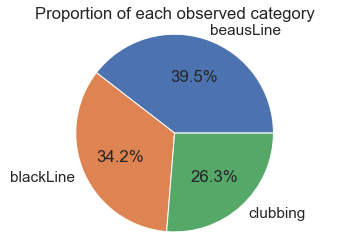

Supplement: Supplemental Information 1 [file peerj-cs-09-1533-s001.zip › Codes and Console Outputs Supplementary Files/Code and Console Outputs for VGG16/Figure 2023-03-09 143103 (1).png]

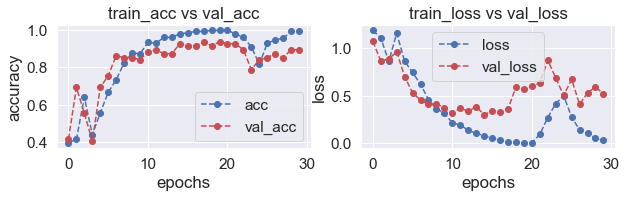

Supplement: Supplemental Information 1 [file peerj-cs-09-1533-s001.zip › Codes and Console Outputs Supplementary Files/Code and Console Outputs for VGG16/Figure 2023-03-09 143103 (10).png]

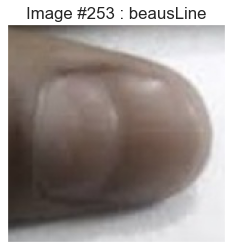

Supplement: Supplemental Information 1 [file peerj-cs-09-1533-s001.zip › Codes and Console Outputs Supplementary Files/Code and Console Outputs for VGG16/Figure 2023-03-09 143103 (2).png]

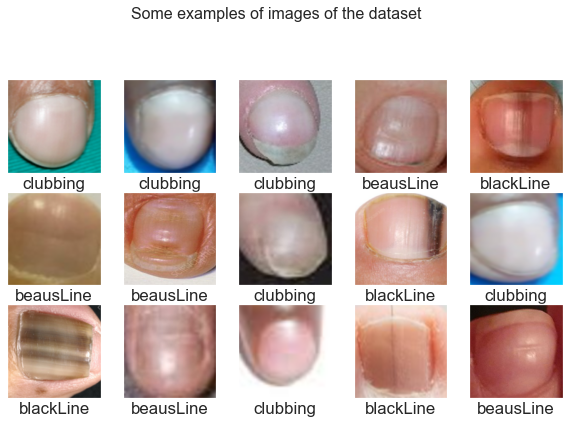

Supplement: Supplemental Information 1 [file peerj-cs-09-1533-s001.zip › Codes and Console Outputs Supplementary Files/Code and Console Outputs for VGG16/Figure 2023-03-09 143103 (3).png]

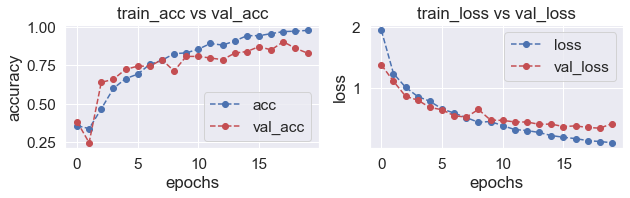

Supplement: Supplemental Information 1 [file peerj-cs-09-1533-s001.zip › Codes and Console Outputs Supplementary Files/Code and Console Outputs for VGG16/Figure 2023-03-09 143103 (4).png]

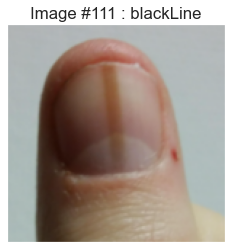

Supplement: Supplemental Information 1 [file peerj-cs-09-1533-s001.zip › Codes and Console Outputs Supplementary Files/Code and Console Outputs for VGG16/Figure 2023-03-09 143103 (5).png]

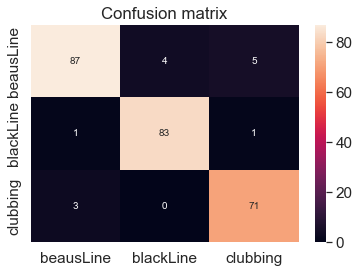

Supplement: Supplemental Information 1 [file peerj-cs-09-1533-s001.zip › Codes and Console Outputs Supplementary Files/Code and Console Outputs for VGG16/Figure 2023-03-09 143103 (6).png]

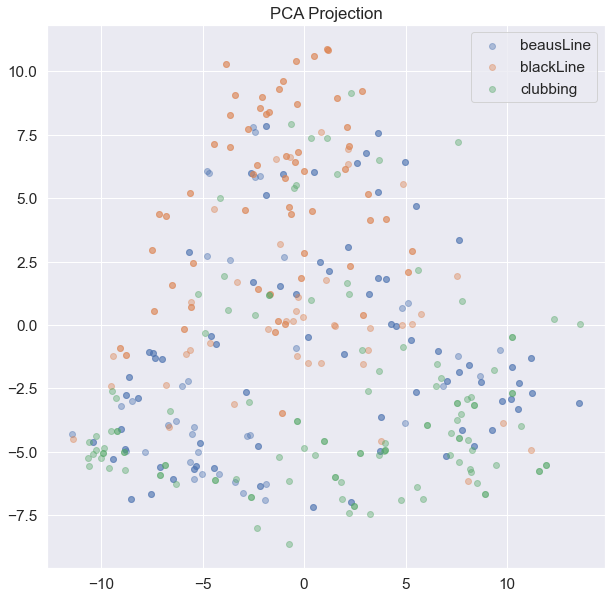

Supplement: Supplemental Information 1 [file peerj-cs-09-1533-s001.zip › Codes and Console Outputs Supplementary Files/Code and Console Outputs for VGG16/Figure 2023-03-09 143103 (7).png]

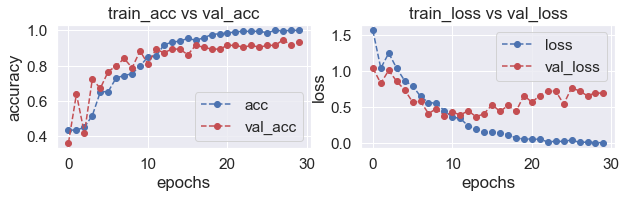

Supplement: Supplemental Information 1 [file peerj-cs-09-1533-s001.zip › Codes and Console Outputs Supplementary Files/Code and Console Outputs for VGG19/Figure 2023-03-09 145153 (10).png]

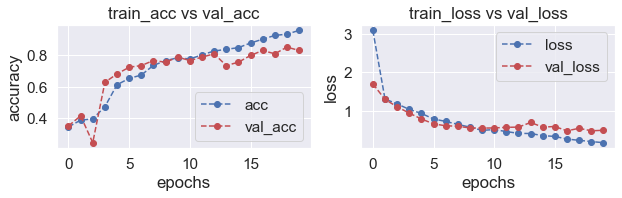

Supplement: Supplemental Information 1 [file peerj-cs-09-1533-s001.zip › Codes and Console Outputs Supplementary Files/Code and Console Outputs for VGG19/Figure 2023-03-09 145153 (4).png]

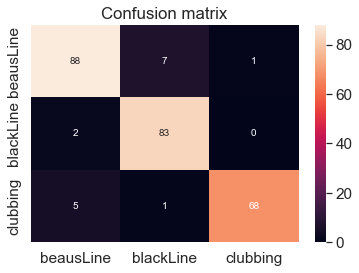

Supplement: Supplemental Information 1 [file peerj-cs-09-1533-s001.zip › Codes and Console Outputs Supplementary Files/Code and Console Outputs for VGG19/Figure 2023-03-09 145153 (6).png]

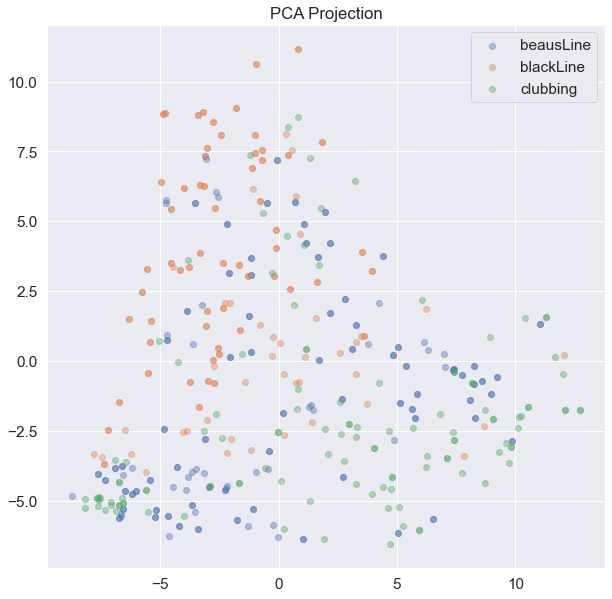

Supplement: Supplemental Information 1 [file peerj-cs-09-1533-s001.zip › Codes and Console Outputs Supplementary Files/Code and Console Outputs for VGG19/Figure 2023-03-09 145153 (7).png]
